# Editorial from the new editor-in-chief

**DOI:** 10.1016/j.jtauto.2023.100224

**Published:** 2023-12-01

**Authors:** Yves Renaudineau

**Affiliations:** Immunology Laboratory Department, Purpan, University Toulouse III, INSERM UMR1291 - CNRS UMR5051, University Toulouse III, Toulouse, France

As the new editor-in-chief of the Journal of Translational Autoimmunity (JTAI), I would like to acknowledge and thank my predecessor, Prof. Eric M. Gershwin at the University of California - Davis, who created and developed JTAI with the vision of a peer-reviewed journal at the front line between basic and clinical research in autoimmunity. During that time JTAI was associated with the Journal of Autoimmunity as a companion journal, with JTAI presenting special and high impact issues. JTAI received its first impact factor in 2023 while expanding its global readership. Without doubt, JTAI is already a well-established journal focusing on translational immunology.

To maintain the highest scholarship for the journal, my goal is for JTAI to promote the importance and visibility of translational research and, towards that goal, JTAI is the appropriate journal to develop a top forum for proposals, discussions and debates regarding the key issues and challenges in translational autoimmunity.

With the help of the editorial board, the aims of JTAI are to publish up-to-date, high-quality and original research papers alongside relevant and insightful reviews in the forefront of translational autoimmunity. Research areas include genetics, epigenetics, cellular, molecular biology, virology, microbiology, omics, immunologic mechanisms, therapeutics, and data obtained from animal models of autoimmunity. Areas of clinical interest are rheumatology, nephrology, dermatology, internal medicine, endocrinology, hematology, and immunopharmacology. Manuscripts that address methods for, and issues related to rigorous translational clinical design, laboratory experiment reproducibility, biomarker comparison, as well as the use of optimized statistical tools will also be considered. I also thank the authors and future authors of JTAI for promoting the highest quality of scholarship.

Please do not hesitate to reach out to me or the co-editors, Drs. Julie Belliere and Wesley H Brooks, with your questions and proposals including for special issues.

